# Intracerebral Hemorrhage Following Bypass Surgery for Atherosclerotic Internal Carotid Artery Occlusion Without Hyperperfusion: The Potential Role of Donor-Recipient Mismatch

**DOI:** 10.7759/cureus.87105

**Published:** 2025-07-01

**Authors:** Yuya Miyata, Satoshi Hori, Katsumi Sakata, Tetsuya Yamamoto

**Affiliations:** 1 Department of Neurosurgery, Yokohama City University Medical Center, Yokohama, JPN; 2 Department of Radiation Oncology, Yokohama City University Hospital, Yokohama, JPN

**Keywords:** atherosclerotic occlusive cerebrovascular disease, hemorrhagic complications, hyperperfusion, sta-mca anastomosis, vessel caliber

## Abstract

Postoperative intracerebral hemorrhage (ICH) following superficial temporal artery to middle cerebral artery (STA-MCA) anastomosis for atherosclerotic occlusive cerebrovascular disease is rare. Hyperperfusion syndrome is considered a primary cause; however, many aspects remain unclear. A case of a 77-year-old man referred for further examination after presenting with left-sided visual disturbance. Magnetic resonance imaging (MRI) showed no cerebral infarction, but MR angiography (MRA) revealed a left internal carotid artery (ICA) occlusion. Single-photon emission computed tomography (SPECT) using N-isopropyl-p-[^123^I]iodoamphetamine (^123^I-IMP) demonstrated that the cerebral blood flow (CBF) value in the left MCA territory was 77% of that on the right side, with a 9.6% increase following acetazolamide challenge. The patient underwent STA-MCA anastomosis to prevent further ischemic stroke. Post-anastomosis, the STA and M4 diameters were 3.1 mm and 1.6 mm, respectively, resulting in a caliber mismatch ratio (STA/M4) of 1.94. Postoperatively, strict systolic blood pressure control (below 130 mmHg) was implemented. However, the patient experienced partial seizures in the left face, while computed tomography (CT) revealed an ICH in the left temporal lobe on the fourth postoperative day. An increase in CBF was not considered to fall within the range of hyperperfusion on ^123^I-IMP SPECT. His symptoms gradually improved with conservative management, returning to a modified Rankin Scale of 1 by the 10th postoperative day. Even in the absence of imaging evidence of hyperperfusion, a marked donor/recipient caliber mismatch may be a potential risk factor for postoperative hemorrhagic complications following direct bypass surgery for atherosclerotic occlusive disease.

## Introduction

Superficial temporal artery to middle cerebral artery (STA-MCA) anastomosis for occlusive cerebrovascular disease is effective at preventing further stroke in patients with reduced cerebral blood flow (CBF). According to the Japanese EC-IC Bypass Trial (JET), cerebral revascularization is indicated for patients with symptomatic internal carotid artery (ICA) occlusion and either MCA occlusion or severe stenosis, when quantitative cerebral perfusion assessment using positron emission tomography (PET) or single-photon emission computed tomography (SPECT) demonstrates impaired cerebrovascular reserve - defined as resting CBF in the MCA territory below 80% of normal and acetazolamide-induced cerebrovascular reactivity (CVR) below 10%. In such cases, surgical revascularization has been shown to reduce the risk of ipsilateral recurrent cerebral infarction compared to medical therapy alone [1]. It is known to provide a sudden increase in blood flow, and although rare, potentially result in intracerebral hemorrhage (ICH). Hemorrhagic complications following revascularization surgery for moyamoya disease (MMD) have been reported in 2.99% to 3.6% of cases, indicating a relatively low incidence [2,3]. In contrast, such complications are exceedingly rare after revascularization procedures for atherosclerotic occlusive disease, with only limited reports available [4].

One well-known cause of this phenomenon is hyperperfusion, which occurs when a large volume of blood flows into a brain region where autoregulation has been impaired due to chronic hypoperfusion. This phenomenon is more commonly observed in cases of MMD [2,5,6], whereas it is relatively rare in arteriosclerotic occlusive disease [4]. Compared to atherosclerotic occlusive disease, MMD is characterized by increased expression of vascular endothelial growth factor and poorer network formation between the pial arteries, which are thought to contribute to the heightened vulnerability to cerebral hyperperfusion [4]. In addition to hyperperfusion, several other potential causes of cerebral hemorrhage following STA-MCA anastomosis have been hypothesized [7]; however, many aspects of their detailed pathophysiology remain unclear. Horie et al. demonstrated that postoperative hyperperfusion following STA-MCA bypass for MMD is associated with a marked mismatch in donor and recipient vessel calibers [8]. Accordingly, a significant caliber discrepancy between donor and recipient arteries may contribute to the development of hemorrhagic complications after bypass surgery.

While perioperative hemorrhagic complications after bypass surgery are uncommon, they can result in serious neurological sequelae and should therefore be avoided. However, the underlying mechanisms remain poorly understood, underscoring the importance of detailed analysis and case sharing. Here, we report a case of atherosclerotic ICA occlusion treated with STA-MCA anastomosis, in which postoperative ICH occurred despite the absence of radiological evidence of hyperperfusion. To the best of our knowledge, no previous reports have demonstrated an association between donor-recipient caliber mismatch and the occurrence of ICH following bypass surgery for atherosclerotic occlusive disease; however, we propose that donor-recipient caliber mismatch may independently contribute to ICH, even without hyperperfusion.

## Case presentation

**Figure 1:**
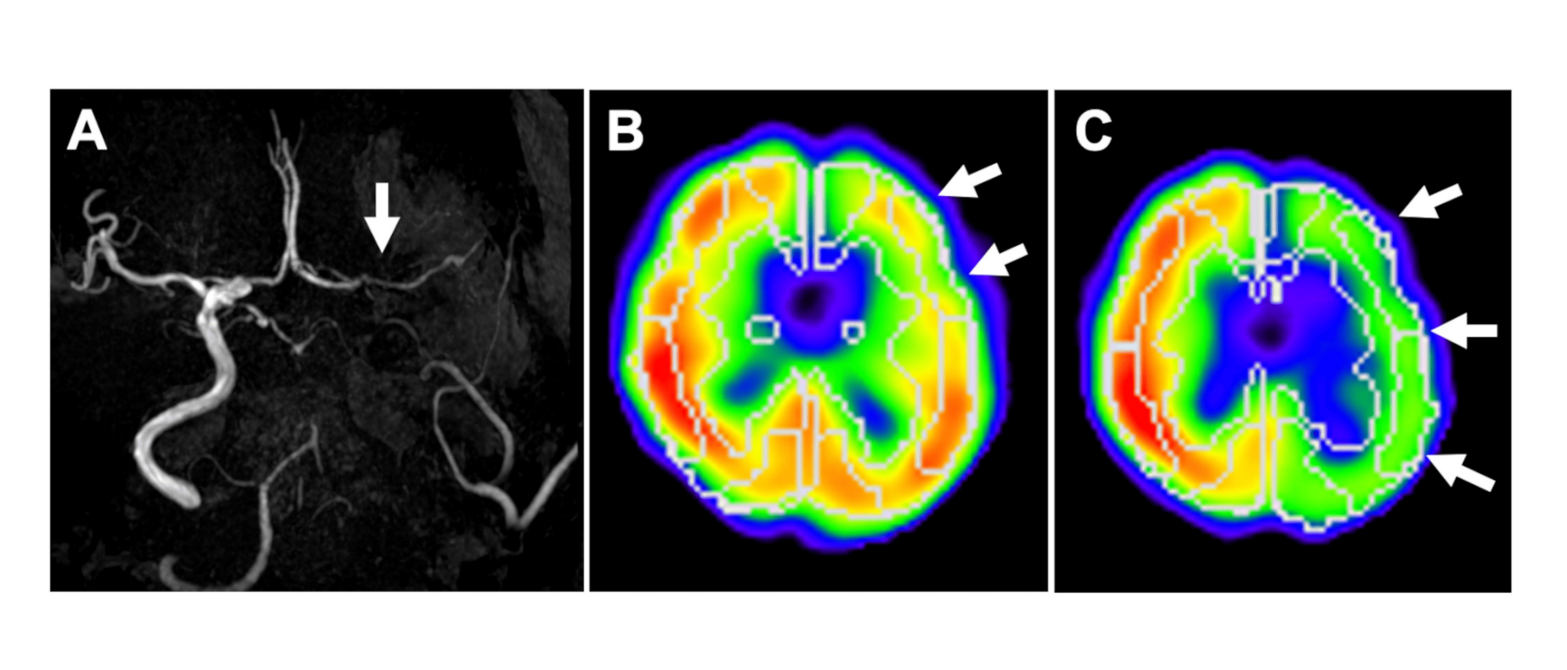
Preoperative magnetic resonance angiography and single-photon emission computed tomography (A) Preoperative magnetic resonance angiography showing the left internal carotid artery (ICA) occlusion (white arrow). (B) Preoperative single-photon emission computed tomography (SPECT) with N-isopropyl-p-[^123^I]iodoamphetamine (^123^I-IMP) was performed to assess cerebral blood flow (CBF) and cerebrovascular reactivity (CVR) to acetazolamide, showing cerebral hypoperfusion (white arrows) and (C) decreased CVR in the left middle cerebral artery (MCA) (white arrows).

A 77-year-old man presented to an ophthalmologist with a sudden decline in visual acuity in his left eye. Central retinal artery occlusion (CRAO) was suspected, and antithrombotic therapy was initiated; however, no improvement in vision was observed. He was subsequently referred to our department for further evaluation to determine the underlying cause of the CRAO. The patient had a history of hypertension and dyslipidemia and was receiving antihypertensive medication, and the systolic blood pressure had been maintained in the 130 mmHg range. Regarding antithrombotic therapy, he was on antiplatelet medication. He exhibited no apparent abnormalities in platelet count or coagulation function. Magnetic resonance imaging (MRI) revealed no obvious cerebral infarctions, and MR angiography (MRA) revealed left ICA occlusion (Figure 1a). SPECT with N-isopropyl-p-[^123^I]iodoamphetamine (^123^I-IMP) was performed one week after the MRI to assess CBF, followed by an acetazolamide-challenged SPECT one week later to evaluate CVR, revealing that the resting CBF in the unaffected MCA territory was 31.82 mL/100g/min, whereas that in the affected side was 24.43 mL/100g/min, representing 77% of the contralateral value. Following acetazolamide administration, the CBF in the affected side increased to 26.77 mL/100g/min, corresponding to a 9.6% increase (Figures 1b-1c).

This case presented with visual impairment due to left CRAO, and was subsequently diagnosed with left ICA occlusion. SPECT revealed that the resting CBF on the affected side was less than 80% of the contralateral side, with a CBF increase of less than 10% following acetazolamide challenge. We considered whether to manage the patient conservatively with antithrombotic therapy alone or to proceed with bypass surgery in addition to antithrombotic treatment. The patient and their family were deeply concerned about the potential recurrence of cerebral infarction. They expressed a strong desire to pursue surgical intervention to improve CBF if such an option existed in addition to medical therapy. Although the patient did not fully meet the eligibility criteria for bypass surgery under JET protocol [1] - specifically with respect to age - they consented to the procedure with the expectation that it would improve cerebral perfusion and help prevent further ischemic events.

**Figure 2:**
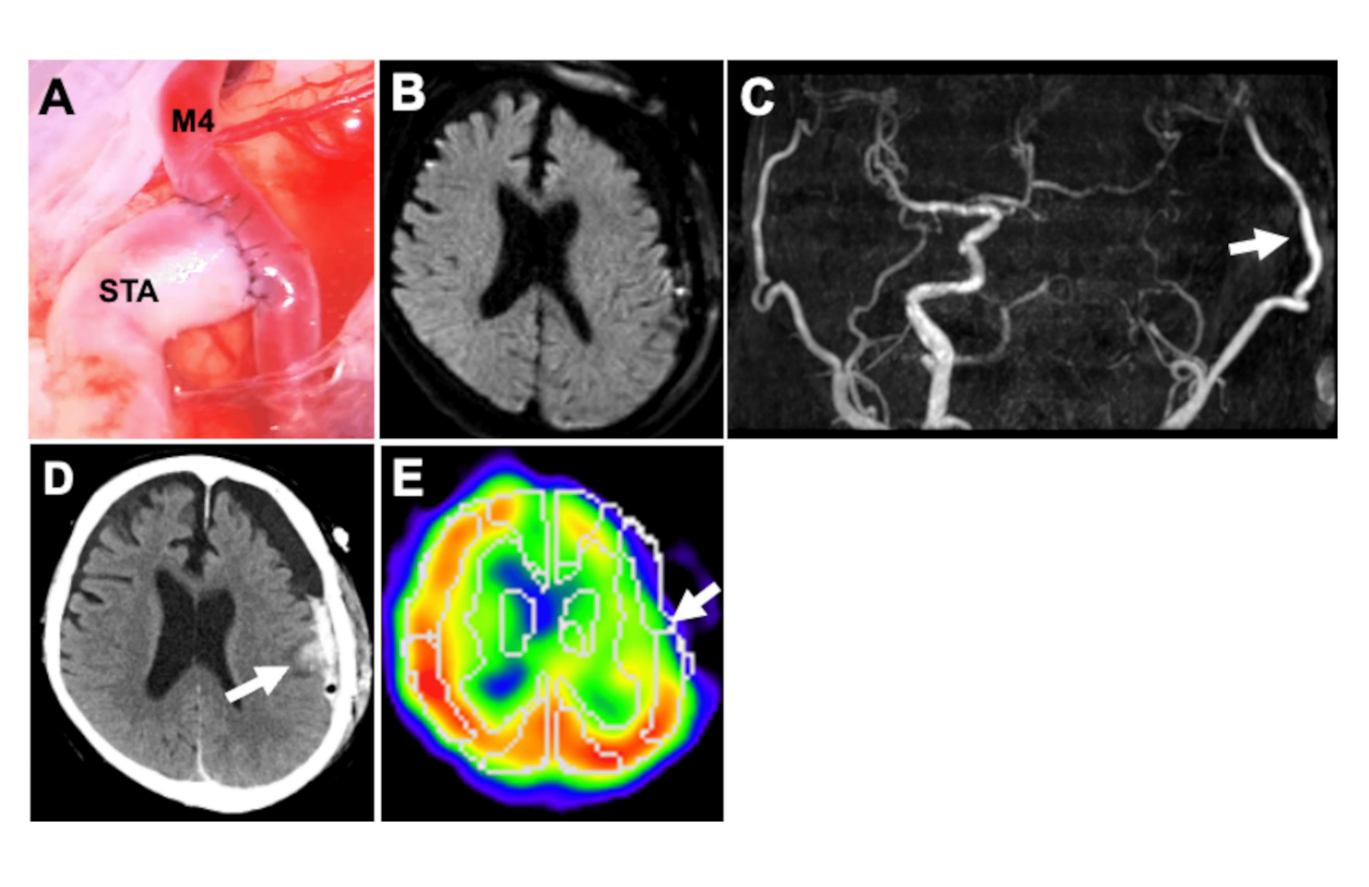
Donor-recipient caliber mismatch and hemorrhagic complication (A) Post-anastomosis, a significant caliber mismatch was observed between the donor STA and the recipient M4. (B) MRI and MRA on day 1 postoperatively revealed no stroke lesions in the brain parenchyma, (C) further demonstrating good bypass patency with strong signals of remarkably thick STA (white arrow). (D) CT on day 4 postoperatively revealed an ICH in the left temporal lobe (white arrow). (E) CBF near the site of anastomosis was not considered to fall within the range of hyperperfusion on ^123^I-IMP SPECT on postoperative day 5 (white arrow). STA: superficial temporal artery; MRI: magnetic resonance imaging; MRA: magnetic resonance angiography; ICH: intracerebral hemorrhage; CBF: cerebral blood flow; ^123^I-IMP: N-isopropyl-p-[^123^I]iodoamphetamine; SPECT: single-photon emission computed tomography

The patient successfully underwent STA-MCA anastomosis of the middle temporal artery to prevent further ischemic stroke. STA was anastomosed to the cortical branches of MCA in an end-to-side fashion with 10-0 nylon threads. The occlusion time of the cortical branch of the MCA during anastomosis was 30 minutes. The patency of the anastomosis was confirmed using indocyanine green (ICG) videoangiography during surgery. Pre-anastomosis, the diameter of the STA was 2.8 mm, while post-anastomosis, the STA and M4 diameters were 3.1 mm and 1.6 mm, respectively, resulting in a caliber mismatch ratio (STA/M4) of 1.94 (Figure 2a). The postoperative dilation of the STA is presumed to result from increased blood flow following its use as a bypass conduit. Regarding postoperative monitoring protocols, postoperatively, the patient was managed in the intensive care unit. Neurological examinations were performed hourly for the first six hours, and subsequently every three hours. As a general protocol, head computed tomography (CT) was conducted immediately after surgery and on the following day, while brain MRI and ^123^I-IMP SPECT were performed within one week postoperatively. When the patient awoke from anesthesia, no new neurological deficits were observed, and the decreased left visual acuity remained unchanged from the preoperative state. Postoperative CT demonstrated no abnormalities. Postoperative blood pressure management in this case was guided by a previous report [9], which demonstrated that maintaining systolic blood pressure below 130 mmHg after STA-MCA bypass for MMD significantly reduces the incidence of symptomatic hyperperfusion. Accordingly, systolic blood pressure was controlled to remain below 130 mmHg postoperatively in the present case. However, motor aphasia suddenly developed on day 1 after bypass surgery; MRI showed no abnormalities in the brain parenchyma, including ischemic and hemorrhagic stroke (Figure 2b), while MRA revealed good bypass patency with high-intensity signals in the remarkably thick STA (Figure 2c). Furthermore, the patient experienced partial left facial seizures on day 4 postoperatively, while CT revealed ICH in the left temporal lobe (Figure 2d). As no hemorrhagic lesions were observed on the immediate postoperative head CT or on the MRI performed the day after surgery, a complication directly attributable to the craniotomy is unlikely. Instead, the hemorrhagic event is considered a postoperative complication associated with the bypass anastomosis. Therefore, the seizure was considered to have been caused by ICH. CBF on the anastomosis site using ^123^I-IMP SPECT on postoperative day 5 was 36.77 mL/100g/min, compared to 47.34 mL/100g/min on the contralateral side, which was not considered to represent hyperperfusion (Figure 2e). Conservative treatment was implemented, consisting of a one-week discontinuation of antiplatelet therapy and strict control of systolic blood pressure to below 120 mmHg, and no expansion of the hemorrhage was observed. Fortunately, the left facial seizure completely resolved by the third day after onset with the administration of antiepileptic medication, and his motor aphasia gradually improved. By postoperative day 10, the patient had returned to the preoperative condition - namely, persistent left visual impairment remained, but he was fully independent in performing all activities of daily living, and the patient was subsequently discharged. Following the onset of motor aphasia, MRI revealed no apparent abnormalities, and given the subsequent resolution of symptoms, the episode was considered a transient neurological deficit associated with the bypass surgery.

A two-year follow-up has been conducted since discharge. Although the patient continues to experience left-sided visual impairment, their ability to perform activities of daily living independently remains unchanged from the time of discharge. No recurrent cerebral infarctions have been observed during the follow-up period. Annual MRI examinations have confirmed that the bypass remains patent and the visualization of the STA is consistently favorable, comparable to the immediate postoperative findings. These results suggest that the bypass has been effective in preventing recurrent ischemic events.

## Discussion

In addition to hyperperfusion, several other factors have been proposed as potential causes of ICH following STA-MCA bypass, including reperfusion injury to previously infarcted and structurally vulnerable brain tissue, damage to perforating arteries branching from the cortical branches of the MCA, and the effects of temporary occlusion of the MCA cortical branch during the anastomosis procedure [7]. In the present case, however, there was no evidence of the former two factors, and the duration of temporary occlusion was approximately 30 minutes, which is within an acceptable range, making these causes unlikely. Furthermore, in cases of atherosclerotic disease, it is assumed that chronic hypertension renders the perforating arteries anatomically fragile. Mechanical stress, such as perioperative blood pressure fluctuations and a sudden increase in CBF due to bypass perfusion, may further exacerbate this vulnerability and contribute to the development of ICH.

To our knowledge, no comprehensive reports have documented cases of ICH occurring after bypass surgery for atherosclerotic occlusive disease in the absence of hyperperfusion findings on cerebral perfusion imaging. In the present case, however, we experienced such an event. This observation raises the possibility that hemorrhage may be attributable to elevated intravascular pressure not necessarily detectable as an increase in CBF. Although such a mechanism has been proposed in MMD, in which recipient vessels are pathologically fragile [3], its applicability to atherosclerotic lesions remains uncertain, and other contributing factors may be involved.

As a potential underlying mechanism for hemorrhagic complications occurring in the absence of radiological evidence of hyperperfusion, we focused on the marked discrepancy in diameter between the STA, serving as the donor, and the cortical branch of the MCA, serving as the recipient. To the best of our knowledge, no previous studies have investigated the association between donor-recipient caliber mismatch and hemorrhagic complications in the context of cerebral revascularization for atherosclerotic occlusive disease. Applying Bernoulli’s principle, a significant disparity between the donor and recipient diameters leads to a significant increase in blood flow velocity on the recipient side, imposing severe localized stress. This localized stress may have led to structural injury of the recipient artery at the anastomotic site, ultimately resulting in hemorrhagic complications. According to Bernoulli’s principle, an increase in the cross-sectional area at the bypass anastomosis results in a corresponding rise in flow velocity on the recipient side, thereby imposing substantial hemodynamic stress. In standard bypass procedures, the donor vessel is typically trimmed in a fish-mouth or oblique fashion; however, to reduce stress on the recipient artery, an oblique cut is considered more favorable than a fish-mouth incision. In the present case, the STA was markedly dilated, and the recipient artery was expected to be subjected to significant stress even under standard conditions. Therefore, to avoid further enlargement of the cross-sectional area at the anastomosis, we shaped the donor stump in an almost perpendicular fashion. Nonetheless, from the perspective of post-anastomotic flow dynamics, such a perpendicular configuration may have been inappropriate. The histopathological findings of MMD differ from those of atherosclerotic occlusive disease and are characterized by fibromuscular intimal thickening without inflammatory cell infiltration or lipid deposition, marked medial thinning, and tortuous and laminar structures of the internal elastic lamina. These features are thought to underlie the vascular fragility observed in MMD. Even in the absence of such structural fragility characteristic of MMD, a marked discrepancy in diameter between the donor and recipient vessels may lead to ICH as a result of localized intravascular pressure elevation.

Since this mechanism is not necessarily related to the overall increase in CBF induced by the bypass, it may occur without radiological evidence of hyperperfusion. However, as neither the pressure gradient nor the flow velocity between the donor and recipient vessels was directly measured, attributing the occurrence of ICH to the caliber mismatch based on Bernoulli’s principle remains speculative and constitutes a limitation of this study. While Bernoulli’s principle is sometimes applied to estimate pressure differentials in human blood flow [10], the complexity of circulatory dynamics in the human body, including cerebral circulation, characterized by the non-negligible elasticity of blood vessels and the viscosity of blood, extends beyond the assumptions of classical fluid dynamics. Therefore, applying Bernoulli’s principle to discuss the relationship between donor-recipient vessel diameter mismatch and ICH is inherently limited.

When performing STA-MCA bypass, clinicians often focus on the risk of postoperative hyperperfusion, a relatively well-recognized complication, with the prevailing notion that hemorrhagic events typically occur as a consequence of hyperperfusion [4-6,8,9]. However, this case highlights the importance of acknowledging that a marked discrepancy in diameter between the donor and recipient vessels may lead to hemorrhagic complications even in the absence of radiological evidence of hyperperfusion. In other words, a substantial caliber mismatch between the donor and recipient vessels may represent a potential predictor of hemorrhagic complications. However, in this case, postoperative SPECT was performed only on postoperative day 5, raising the possibility that early postoperative changes in hyperperfusion may have been missed. This represents a limitation of the present study.

On the other hand, Horie et al. reported that a marked discrepancy in diameter between the STA and MCA was significantly associated with the development of postoperative hyperperfusion following STA-MCA bypass in patients with MMD [8]. The authors demonstrated an association between donor-recipient caliber mismatch and the occurrence of postoperative hyperperfusion exclusively in adult cases of MMD, whereas no such correlation was observed in pediatric cases, suggesting that caliber mismatch alone is unlikely to be the sole determinant of hyperperfusion. Furthermore, in their atherosclerotic occlusive disease cases, despite a relatively high median donor-recipient mismatch ratio exceeding 1.5, no cases of postoperative hyperperfusion were reported. These observations imply that, in cases of atherosclerotic occlusive disease such as our case, the presence of donor-recipient caliber mismatch may not necessarily result in hyperperfusion. However, when the mismatch ratio exceeds 1.9, as observed in our case, it may pose a potential risk for ICH, even in the absence of overt hyperperfusion. Therefore, such anatomical conditions nonetheless highlight the importance of meticulous postoperative management.

## Conclusions

We experienced a case of postoperative ICH following STA-MCA bypass for atherosclerotic ICA occlusion, despite the absence of radiological signs of hyperperfusion. While clinical attention is commonly directed toward hemorrhagic complications associated with postoperative hyperperfusion, it must be acknowledged that such complications can arise even in the absence of detectable hyperperfusion following bypass surgery. A diameter mismatch between the donor and recipient vessels may represent a potential risk factor for postoperative ICH following bypass surgery. However, this report is based on a single case, and definitive conclusions cannot be drawn without larger-scale studies involving more cases in the future. Furthermore, the postoperative ICH in this case may also have been a complication related to the craniotomy itself. Postoperative management should include strict blood pressure control and vigilant monitoring for any neurological changes, with immediate imaging evaluation if such changes are observed, in anticipation of the potential for hemorrhagic events.
